# Influence of Sandblasting Particle Size on the Shear Bond Strength of Orthodontic Brackets to Milled and 3D-Printed Provisional Crowns or Materials After Artificial Aging

**DOI:** 10.3390/jfb16120457

**Published:** 2025-12-08

**Authors:** Khurshid Mattoo, Mohammed E. Sayed, Marwan Someli, Ahmed Alhazmi, Mohammed Khawajy, Shroog A. Almasoudi, Ebrahim Fihaid Alsubaiy, Saeed M. Alqahtani, Mohammed A. Alfaifi, Raghdah M. Alshaibani

**Affiliations:** 1 Department of Prosthetic Dental Sciences, College of Dentistry, Jazan University, Jazan 45142, Saudi Arabia; drsayed203@gmail.com; 2 Intern Clinics, College of Dentistry, Jazan University, Jazan 45142, Saudi Arabia; mmm.sumaily@gmail.com (M.S.); ahmad.094122@gmail.com (A.A.); abu.prins14111@gmail.com (M.K.); shroog.ali.j@gmail.com (S.A.A.); 3 Department of Prosthetic Dental Sciences, College of Dentistry, King Khalid University, Abha 62529, Saudi Arabia; alsbuay@kku.edu.sa (E.F.A.); smaalqahtani@kku.edu.sa (S.M.A.); mualfaifi@kku.edu.sa (M.A.A.); 4 Department of Clinical Dental Sciences, College of Dentistry, Princess Nourah Bint Abdulrahman University, P.O. Box 84428, Riyadh 11671, Saudi Arabia; rmalshaibani@pnu.edu.sa

**Keywords:** dental crowns, three-dimensional printing, computer-aided design, orthodontic bracket, temporary dental restoration, provisional crowns, provisional composite materials, fixed partial denture, multidisciplinary treatment

## Abstract

Purpose: This in vitro study ascertained the impact of three distinct alumina particle sizes on the shear bond strength (SBS) between two distinct provisional crowns (milled and 3D-printed) and stainless-steel orthodontic brackets following artificial aging. Materials and methods: Eighty specimens [disc 10 mm diameter/15 mm height] were fabricated with two provisional crown materials, milled (CopraTemp) [group (GP) M] and three-dimensionally printed (Asiga DentaTooth) (GP P), and divided into eight subgroups based on alumina oxide (sandblasting) particle size surface treatments of 25 μm [P25, M25], 50 μm [P50, M50], and 100 μm [P100, M100], with no surface treatment specimens serving as control [PC, MC]. After thermocycling (2200 cycles), the SBS and Adhesive Remnant Index (ARI) were calculated. Statistical tests included one-way analysis of variance (ANOVA) (Kruskal–Wallis), followed by post hoc tests [Tukey HSD, Dunn’s], with the probability ‘*p*’ value being significant at 0.05 (*p* ≤ 0.05). Results: Without surface treatment, the 3D-printed provisional crown had the lowest SBS [median (IQR); 12.8 (2.74)]. The highest SBS was found in both milled and 3D-printed PMs with 50-micron particle sizes [Milled = 23.10 (2.3); Printed = 20.72 (2.31)], followed by 100-micron [Milled = 20 (2.36); Printed = 17.99 (3.45)] and 25-micron [Milled = 16.13 (2.71); Printed = 15.08 (1.55)]. The majority of cohesive failures were seen in the milled subgroups, while all subgroups of 3D-printed provisional material had adhesive bond failures. Conclusions: Sandblasting, irrespective of particle size, enhances SBS in both milled and 3D-printed provisional restorations; however, 50-micron alumina particles are recommended since they enhance SBS substantially.

## 1. Introduction

Orthodontic dental treatments, especially those that involve translatory tooth movements, involve long treatment times, varying from several months to several years [1]. Use of a provisional/temporary crown during the course of orthodontic treatment is desirable because definitive crowns/restorations are fabricated once orthodontic correction is completely accomplished [2]. Provisional crowns during orthodontic treatment are indicated in cases of root extrusion, for aesthetic maintenance, tooth movement prevention, stabilization, and pre-restorative orthodontic treatment. They prevent teeth from becoming sensitive, keep the coronal seal intact, and stop teeth from shifting. They are also of significant value in orthodontic treatment for young patients and children, where crowns cannot yet be given due to incomplete or ongoing tooth eruption and ever-changing gingival architecture. Debonding of any type of orthodontic bracket (stainless steel, ceramic, or plastic), irrespective of being bonded to a natural tooth or a provisional crown, disrupts the influence of applied forces, which delays treatment goals, thereby being a source of frustration for both the clinician and the patient [3]. Clinically, shear forces are the worst forces that can affect an orthodontic bracket bond, while forces that are tensile or torsional in nature are not so detrimental for debonding [1,4]. Shear bond strength (SBS) is a critical indicator of adhesion quality in all types of dental restorations since they are weaker compared to natural tooth structure like enamel. High SBS minimizes interfacial failure, thereby increasing restoration longevity. Factors reducing SBS to tooth enamel include substrate and surface factors like smear layer presence, caries-affected dentin, and porcelain and enamel conditioning. However, some of these factors play a lesser role for restorative materials, where SBS depends largely on the material itself. Shear bond strength is a biomechanical index for functional loading adhesive performance. It predicts and optimizes restorative, orthodontic, and biomedical bond durability using experimental testing and computational modeling. Different kinds of clinical forces must be applied for orthodontic tooth movements; for instance, extrusion requires 35 to 60 g, whereas tooth translation or body movements require higher forces in the range of 70 to 120 g [1,5]. It has been established that during orthodontic treatment, the long-term shear bond strength to natural enamel must be between 6 and 8 MPa [1,5]. Since such testing has been primarily determined in laboratory experiments, many clinicians and researchers have questioned these threshold values based on the clinical variables that play significant roles in influencing the bracket bond strength [1,6]. These clinical factors include the oral cavity’s varying potential of hydrogen (pH), varying temperature and humidity depending upon the food intake type, adhesive fatigue, and bacterial degradation associated with orthodontic bracket adhesives and patients’ dietary habits [6,7,8]. Reports that have found in vivo-aged bond strength to be much lower than in vitro-aged bond strength [4,8] back this up. There are other influences upon clinical bond strength besides the differences between in vivo and in vitro environments. These include the type of provisional material used, the type of adhesive used, the length of time the material is stored, and the age of the restorative material. Clinical failure rates of brackets range from 0.5% to 55.8% [9], with mandibular molars having higher failure rates (2.7 to 29%) [10]. Peak failure occurs after placement and after treatment, with early loss often due to inadequate clinical technique [1]. Shear bond strength of orthodontic brackets has been observed to vary according to the substrate, which has included tooth enamel [11,12], restorative or filled resins [13,14,15], dental ceramic substrates [16,17], silver amalgam [1,18], unfilled methacrylate-based provisional resins [19,20], and filled or composite-based provisional resins [13,21,22,23]. Orthodontists attach brackets to temporary composite crowns and use provisional crowns for long periods of time during treatment. This provides temporary protection, stability, and function while they figure out how the final restoration will look [24].

FDA regulations recommend four key dental materials for provisional restorations, namely, composite resins (bisacryl composite, light-cure composite), polymethylmethacrylate (PMMA), and polyethylmethacrylate (PEMA) [1]. While bisacryl and light-cured composites have largely replaced the polymethylmethacrylates due to added advantages [better marginal adaptation, higher strength, better color stability, less shrinkage], digital advances like computer-assisted diagnosis and computer-assisted machining (CADCAM) have seen a resurgence of PMMA and PEMA-based provisional resins since major drawbacks associated with PMMA provisional restoration manufacturing, like shrinkage, exothermic reaction, and degradation of the matrix, have been overcome either through milling of a pre-polymerized PMMA puck [19,20] or via three-dimensional PMMA resin ink printing [1,25]. Aesthetic brackets made of either ceramic [26] or plastic [27,28] have further renewed researchers’ interest in determining the shear bond strengths to different digitally fabricated provisional materials using different surface treatments. Stainless-steel orthodontic brackets, despite having a high elastic modulus, continue to be popular due to high stiffness, resilience, biocompatibility, resistance to intraoral corrosion, and higher strength [4,29]. The surface treatment also greatly influences the bond strength of orthodontic brackets. Techniques include mechanical (sandblasting, grinding with diamond burs) and chemical (etching with phosphoric acid and hydrofluoric acid) approaches [1,4,5,6,29,30]. A popular, effective, and clinically applicable method is that of air-blasting using alumina or sand particles on all types of orthodontic brackets and provisional restorations [5,6,12,19,20,22]. Another significant clinical influence on the shear bond strength is the aging that the bracket and the provisional restoration or the crown will undergo [1,4,12,20,21], while in some clinical cases, the restoration may already be aged and therefore influence the SBS of the orthodontic bracket [13,26,29]. In both situations, the bond strength has been reported to be significantly improved by different surface treatments.

Milling and 3D printing technology are currently being utilized for various restorative materials, including resins (composites, polymers), dental ceramics, and metals/alloys, for making either temporary or permanent restorations, dental casts and models, dental implant-supported prostheses, and various maxillofacial prostheses [31,32,33]. Milled pre-polymerized resin pucks provide superior strength, durability, aesthetics, and enhanced mechanical properties for provisional restorations after aging; however, their high cost and the increased waste, wear, and heat production associated with them make these pucks a costly clinical alternative that is also less eco-friendly [34]. Three-dimensional printing has recently become increasingly popular, especially for customized uses, utilizing techniques like material jetting, stereolithography, digital light processing, and material extrusion [35]. Three-dimensional printing offers advantages to a dental clinician like minimal material use (economical and eco-friendly), low cost (raw material), and high resolution for intricate details, while initial disadvantages like shrinkage and surface roughness can be overcome by proper calibration [1,36]. Studies have shown that provisional crowns fabricated via 3D printing have superior internal crown fit [37] and marginal fit [38] and higher wear resistance [39]. Printer optimization in terms of orientation and print thickness has been shown to enhance physical and mechanical properties of the final product [40]. Compared to PMMA resin (heat cure), a printed resin (Asiga Dentamodel) was found to have lower surface roughness (0.19 μm), resulting in improved long-term durability in optical and mechanical properties [41], which are essential requirements for provisional crowns. Few studies have investigated the SBS between orthodontic brackets and milled or 3D-printed provisional crowns. Haber D et al. [19] reported that orthophosphoric acid etching should not be used on milled CAD/CAM PCs and that mechanical surface treatments and chemical treatments produce SBS equivalent to natural teeth. Goracci et al. [20] recommended using a medium grit and universal adhesive to increase the SBS between metal brackets and PMMA CADCAM PCs. In another recent study [42], flowable composites, when bonded with either ceramic or plastic brackets, had higher SBS than packable composites, while ceramic brackets had higher SBS than plastic brackets for both flowable and packable composites. Hassan NN et al. [1] compared SBS between stainless-steel orthodontic brackets and milled and 3D-printed provisional crown restorations while also reporting sandblasting to have a higher influence than coarse and fine diamond surface preparation. The study used only one particle size, which was 50 μm, with bond strength increasing in both milled and 3D-printed provisional crown resins. Most intraoral sandblaster units provide an option of using alumina particle sizes between 20 and 70 microns, while particle sizes higher than these are generally utilized in production laboratory-based sandblasters. However, there are no clear recommendations as to which particle size a clinician should use while performing mechanical surface treatment on milled and 3D-printed provisional crowns that enhances SBS to the maximum. The goal of our innovative research is to fill this knowledge gap and provide practical suggestions for treatment. Therefore, this study was primarily aimed at determining the influence of three different alumina particle sizes (25 μm, 50 μm, and 100 μm) on the SBS between stainless-steel orthodontic brackets and two different provisional crowns (milled and 3D-printed) after artificial aging of 2200 cycles, which is equivalent to 18 months of clinical time. The objective of this study is to determine which particle size produced a consistent increase in SBS and if there is any need for a clinician to perform surface sandblasting using higher particle sizes. We hypothesize that milled and 3D-printed materials will have different SBS and particle sizes will affect SBS, while an alternate hypothesis would state that materials and particle sizes will not affect SBS.

## 2. Materials and Methods

Ethical approval: This in vitro study was proposed as student research for the academic year 2024–2025 to the ethical committee at Jazan University, who approved the same under registration/reference number CODJU-2424I. This study did not require taking any informed consent since no patients or participants were involved.

**Figure 1:**
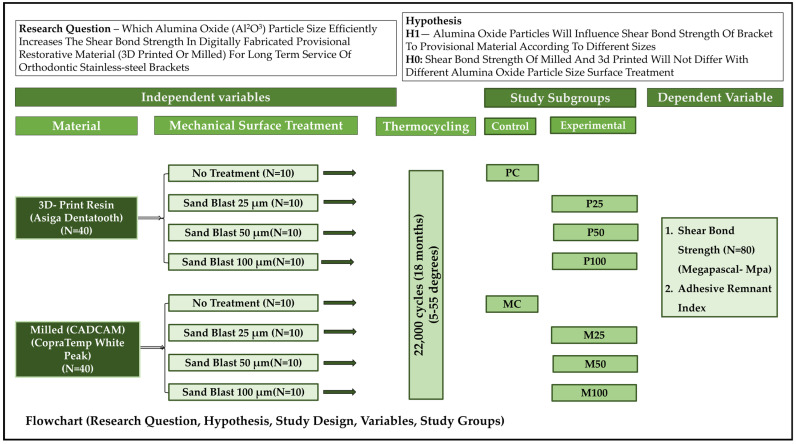
Flowchart showing the overall study design, hypothesis generated, the independent and dependent variables, and the study groups.

Study design: With provisional materials, surface treatments, and aging serving as independent variables, this study was designed to follow a comparative approach between a set of control and experimental groups. Sample preparation represented the initial phase, which was followed by the second phase of bonding and aging, followed last by the testing phase. The study sequence followed is represented in Figure 1, which also depicts the hypothesis and different subgroups.

Operational terms [43]: ‘shear bond strength’ was denoted by the maximum force that could be tolerated by an adhesive interface before failing or fracturing either cohesively or adhesively. ‘Shear stress’ is defined as an internally generated stress or force that opposes a twisting action or the sliding of one plane on an adjacent plane. The term “bonding” refers to a clinical process in which two surfaces are attached with the aid of an intervening adhesive, which in this case is called a bonding agent. A bonding agent is a substance that helps two substances stick together or stay put or helps a material adhere to natural tooth structures.

Sample size: Based on the number of control and experimental groups, the total number of samples was determined using an arithmetical formula (N = 2 σ2 × (Z α + Z β) 2/2), operated through a computing software (Nquery, v7.0; Informer Technologies, Los Angeles, CA, USA). Using guidelines for in vitro research [44], the power assumption was kept as 80%, the effect size (D2) as 0.30, and type 1 error rate as 0.05. With each subgroup having 10 samples, a total of 80 samples (40 milled, 40 printed) were considered to be the minimum for this study. If any sample attained damage or was defective, it was replaced with an extra sample that was kept as spares for each subgroup.

Sample preparation: Table 1 shows a list of experimental materials, including their specifications, features, and manufacturers. The provisional restorative material for milling used was CopraTemp (WhitePeaks Dental Solutions GmbH, Wesel, Germany), while the 3D-printed resin was Asiga Dentatooth (Alexandria, Australia). Both materials are to be used through CADCAM technology. By scanning [desktop scanner (3Shape, Copenhagen, Denmark)] a previously manufactured stainless-steel metal die with comparable dimensions, a disc with precise measurements (10 mm diameter/15 mm height) was created digitally. The data of the digital sample was then transferred to the respective production machines [milling (EXOCAD, Darmstadt, Germany), 3D-print (Asiga, v 2.0)] to yield respective specimens representing provisional crowns or restorations. PMMA resin pucks (CopraTemp, Wesel, Germany) (size 98/14, Shade A3) were dry milled (5-axis) (DWX-52D Series, Roland DGA, Irvine, CA, USA) using standard operating (6 to 1800 mm/min) and spindle speed (6000 to 30,000 rpm) as per the manufacturers’ recommendations [45]. After finishing the milled specimens with the manufacturer-recommended kit, they were polished for 90 s using a polishing slurry (Pumice Fine; Benco Dental, Pittston, PA, USA) [46]. The samples for 3D printing were prepared from Shade A3 resin ink (Asiga DentaTooth, Australia) using a digital light processing printer (Asiga, Australia) at an optimized setting [65 μm pixel size, 4.6 × 2.6 × 3 inches build volume] [1,40]. After printing, the disk was cleaned on its surface with 98% pure isopropyl alcohol in a ventilated room. Two minutes were spent in an ultrasonic cleaner both before and after washing. After being cleaned, each specimen was left for 30 min to ensure that it was alcohol-free. Curing was performed for each sample by exposure to a light source with a wavelength of 385 nm. Following curing, the specimens were cooled and then exposed to 2000 flashes twice, for a total of 4000 flashes—2000 flashes on each side. Fresh water soaking at room temperature completed the washing of the specimens, followed by recommended finishing and polishing using routine adjusting rotary instruments. A plastic ring was then fastened to each sample with the help of self-curing resin, which held the specimen in the desired angular position at the time of testing.

**Figure 2:**
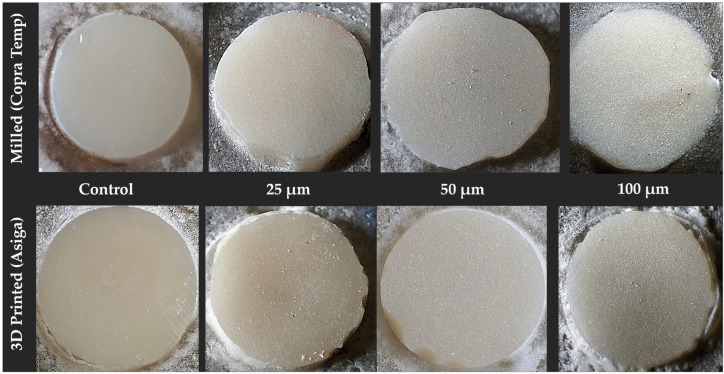
Disk-shaped specimens of two main provisional materials (milled and printed) and their respective control and experimental groups (from left to right): control—no treatment, sandblast (25 μm, 50 μm, and 100 μm Al_2_O_3_).

Mechanical Surface Treatment (Figure 2): For each material subtype, the control group for printed (P) and milled (M) specimens consisted of those that did not receive any surface treatment (PC, MC). The other three intervention groups were characterized by surface treatments using alumina oxide particles of sizes 25 μm (P25, M25), 50 μm (P50, M50), and 100 μm (P100, M100). A single sandblaster that would deliver three different particle sizes was chosen for this study. The sandblaster was first calibrated for air pressure (2–6 bar), particle size (three different chambers for storage and three color-coded nozzles for delivery), and angle of delivery (the nozzle handle fixed onto a stand keeping the nozzle perpendicular to the surface, which requires blasting). Calibration for air pressure was carried out using a pressure gauge, while for particle flow, the system was run briefly before performing the actual procedure so that residual debris and static charges would be flushed out.

With the recommended distance of 10 mm, bar pressure of 3, time duration of 15 s, and the nozzle of the intraoral sandblaster (Microetcher IIA, San Ramon, CA, USA) placed perpendicular to the sample surface, sandblasting of each group was carried out by a single calibrated operator. To accomplish sandblasting of 100 μm, a laboratory sandblaster unit (BeGo, Bremen, Germany) was used while maintaining the same parameters and standards.

Chemical surface treatment (bonding of orthodontic brackets): A total of one hundred similar stainless-steel prefabricated mandibular second premolar orthodontic brackets (Damon2; ORMCO, Glendora, CA, USA) with designated surface areas were used to be bonded with provisional restoration materials. The disclosed surface area of orthodontic brackets by manufacturers was first verified on 15 randomly selected orthodontic brackets and found to be correct [surface area 11.10 mm^2^]. A single light-cure hydrophilic bonding agent (Assure Plus) was used for all samples in all groups. The position of orthodontic brackets was standardized by using a transparent thermoplastic mold that was customized to fit the specimen’s outer ring [1]. A number eight scaler tip (KaVo Perio Tip) was used to firmly seat the bracket once it was in its final position. The disk samples were each coated with a single layer of bonding resin using the recommended micro brush. The resin was then diluted and dried with a stream of dry air. Light curing the adhesive is only advised if the resin used to attach the bracket is dual cure, according to the manufacturer’s guidelines. If the resin used to attach the bracket is light cure, then both can be light cured simultaneously. Concurrently, transbond XT adhesive was applied to the SS bracket base and pressed gently against the specimen’s specified center. Once excess was extruded from the bracket base, it was removed with the same scaler tip that pressed the bracket against the provisional crown material. After removal of excess, the bracket placed on the disc with adhesive paste was cured with LED light (Ortholux Luminous; 3M Unitek), with a standard power of 1000 mW/cm^2^, for a period of 10 s. With a single calibrated operator performing the entire bonding process, the tip of the light cure for all specimens was standardized in terms of distance (10 mm) from the specimen surface and the time of exposure [12 s each on two predetermined sides]. Primer application by some manufacturers of orthodontic bracket adhesive is recommended only for natural tooth enamel and not restorative material.

Artificial aging: To ensure that the in vitro specimens undergo clinically equivalent aging of 20 months of orthodontic treatment, the specimens in each subgroup were placed in a thermocycling machine (SD Mechatronik, Germany), which immersed the samples alternately in a bath of cold and hot water with a temperature range between them from 5 to 55 degrees centigrade for a total of 2200 cycles [19,47]. The immersion time for each lasted 30 s, while the interval between the two corresponded to the interval of 10 s during which the samples were kept in open air.

**Figure 3:**
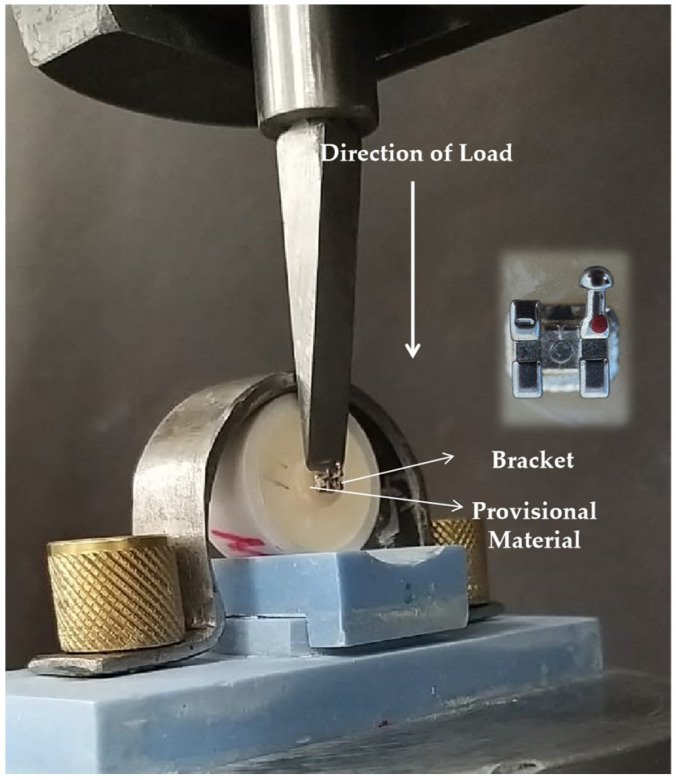
Application of load in relation to the specimen placement and the bonding interface between the orthodontic bracket and provisional restoration material.

Measurements (Figure 3): The shear bond strength for all specimens in each subgroups were evaluated by mechanically testing the specimen-ring assembly with a bonded orthodontic bracket on a universal (Instron, Grove City, PA, USA) test machine. The machine required each specimen to be fastened in such a way that the flat-ended steel rod through which force application is made has its flat end at the junction between bracket and specimen, or in other words, the specimen base remains parallel to the direction of the force. The clamp of the lower portion of the machine held the ring of the specimen, while the flat rod moved at a slow cross speed of 1 mm per minute. The moment the bracket would debond from the specimen, it would break the circuit, and the machine would automatically come to a stop while at the same time showing the values of the failure load. The failure load at which the brackets debonded from the provisional material disk was expressed in newtons, which was later recalculated and expressed in megapascals. The denominator used for such derivation was the surface area of the bracket [Force (N)/Area (m^2^) × 10^−6^].

Adhesive remnant index: Adhesive failure was designated if separation of the adhesive layer from one or both substrates occurred, resulting in the adhesive detaching from the surface and leaving a clean, bare substrate. Cohesive failure was designated if and when the adhesive material broke or split with the substrate, resulting in the adhesive remaining on both surfaces after separation. The surfaces of all the debonded orthodontic bracket to provisional restoration specimens were inspected [magnification 20×] using an optical microscope (Amscope, Irvine, CA, USA). The adhesive remnant index (ARI) examined and analyzed the bracket failure on the specimen by scoring each specimen based on the amount of leftover adhesive [1,42]. Depending upon the percentage of leftover adhesive, scores from 0 (no adhesive) to 3 (75 percent or above adhesive left) were given to each sample. The index provides scores for the failures. To do this, the area was divided into four equal portions, with each section representing a quarter of a whole. From what we could tell under the microscope, there were three distinct types of failure: adhesive failure, in which the adhesive or substrate separated from the substrate; cohesive failure, in which the adhesive itself failed; and mixed failure, in which the adhesive and substrate or substrate and bracket were both partially adhered, or a mix of the two. The amount of adhesive that remained on the sample surface indicates the clinical time required for cleaning the tooth surface after debonding the orthodontic bracket on a patient.

Statistical Analysis: All raw data was recorded in individual Microsoft Excel sheets, following which the data was visualized, refined, and then organized by the respective subgroups for analysis. The values of newtons were transferred into megapascals for each specimen, following which the data was entered into the Statistical Package for Social Sciences (SPSS, V23, IBM, Armonk, NY, USA). Individual subgroup data was checked for distribution (normality Shapiro–Wilk test), followed by the Levene test to verify the homogeneity of variances among different groups. The normality test results suggested deriving the median with interquartile ranges and using a non-parametric test for finding differences between subgroups. A one-way ANOVA (Kruskal–Wallis) rank test determined the differences between two material subgroups and within each material group. Median, interquartile range, and mean rank scores were used to analyze the degree of variances for each subgroup. For the purpose of analyzing the differences in the sample SBS medians between the various subgroups of milled and printed PC groups, a Tukey HSD (Honestly Significant Difference) post hoc pairwise comparison test (Dunn’s test) was utilized. Bonferroni’s correction was utilized to correct the probability value for the post hoc test by using the formula [corrected α = α/m, where α was the value of significant and m was the number of paired subgroups]. For the purpose of statistical analysis, the probability ‘*p*’ value was deemed to be significant if the difference was found to be less than or equal to 0.05 (*p* < 0.05). The adhesive remnant index scores were expressed in frequency distribution (percent) to identify major bond failure related to a particular subgroup.

## 3. Results

Shear bond strength: A comparison of the means of SBS produced from SS orthodontic brackets and two different types of PC materials is shown in Table 2, together with the effects of various surface treatments. A lower SBS was obtained for both controls, with printed showing the lowest [median (IQR); 12.8 (2.74)], which indicates that in the absence of mechanical surface treatment, the milled PR shows higher bond strength than 3D-printed. Alternately, these differences indicate that aging has less effect on milled than printed when it comes to maintaining adequate SBS for long-term duration. When compared to the control group, sandblasting (alumina oxide) increased the SBS for both materials, irrespective of the particle size, thus indicating sandblasting to be an effective way to enhance SBS between orthodontic brackets and long-term provisional restorations. The highest SBS was obtained in both milled and 3D-printed PMs, with 50-micron particle sizes [milled = 23.10 (2.3); printed = 20.72 (2.31)], followed by 100 microns [milled = 20 (2.36); printed = 17.99 (3.45)] and least by 25-micron [milled = 16.13 (2.71); printed = 15.08 (1.55)] particle sizes. This indicates that the particle size used for sandblasting influences the SBS of stainless-steel orthodontic brackets for both milled and printed provisional crown material. The one-way ANOVA test results with the respective degree of variance and their relative significance by ranking are presented in Table 2. The results indicate that the differences between the experimental and their respective control groups were significant when both material subgroups were considered. The ANOVA test results for subgroup comparison within each material were also found to be statistically significant (*p* < 0.05). The results thus implicate that sandblasting, irrespective of particle size, produces significant changes in SBS after aging of both milled and 3D-printed PCMs. The results of the post hoc (Tukey HSD) pairwise comparison test are displayed in Table 3 (both material subgroups) and Table 4 (individual material subgroups), which reflects the pair of subgroups that differed from the other subgroups. The changes brought by different particle sizes of alumina varied but presented a similar picture in both materials. The SBS did not change significantly with 25-micron particle size for both materials when compared with the control (no treatment), and the 50-micron particle size produced SBS changes that were not only significant to the control but also to that of 25 microns for both material subgroups. For both materials, 100-micron particle size produced significant differences from that of the control but did not differ significantly from that of 25- or 50-micron particle sizes in both materials. These results indicate that for both materials, a 50-micron particle size of alumina produces a superior and durable bond with both types of provisional crown materials. Table 4 presents Dunn’s test results of four different subgroups within each individual material, after Bonferroni’s correction for alpha (*p* < 0.0083). For both materials, the results indicate that 25-micron particles did not significantly alter the shear bond strength (SBS) compared to the control, while 50-micron particles caused significant changes in SBS relative to both the control and 25-micron particles. In contrast, 100-micron particles resulted in a significantly different SBS from the control but were not significantly different from either 25- or 50-micron particle sizes. The overall results thus indicate that 50-micron particle alumina oxide sandblasting produces the strongest bond over a period of 20 months or 2200 cycles.

Peak loads and bond failure: Among all subgroups, Gp M50 showed the highest peak loads, followed by the Gp M100 and Gp P50 specimens. However, more specimens in Gp M100 attained peak load when compared to specimens in Gp P50. The lowest peak loads were observed in Gp PC and Gp MC. The patterns for intermediate load among various subgroups show that there are regular variations, with more variations observed in subgroups of 3D-printed than milled provisional crowns. The elastic region, or the linear region, was longer in 3D-printed subgroups than milled subgroups, with slopes being more gradual in milled than 3D-printed specimens, thus indicating the bonds of 3D-printed provisional resin to be stiffer. Bonds associated with 3D-printed provisional crowns also showed earlier plastic deformation than bonds associated with milled subgroups.

**Figure 4:**
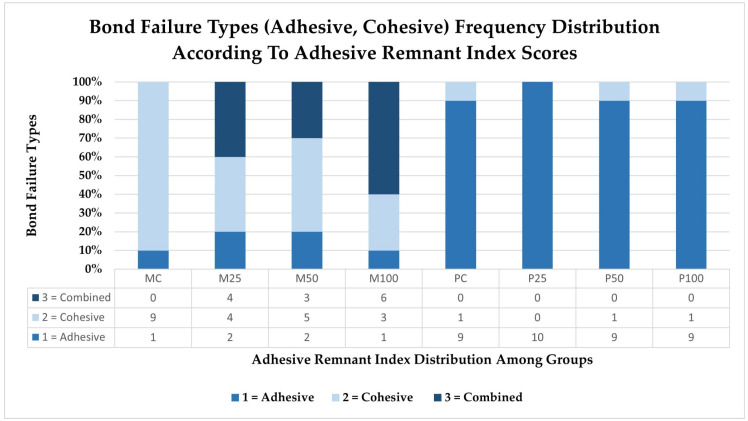
Comparative frequency distribution (in percent) of various bond failure types (adhesive, cohesive) and their respective graded scores (0 = no adhesive, 1 = ≤50%, 2 = ≥50% to 75%, 3 = all adhesive) as per the adhesive remnant index, in specimens of various subgroups of milled and 3D-printed provisional crowns.

**Figure 5:**
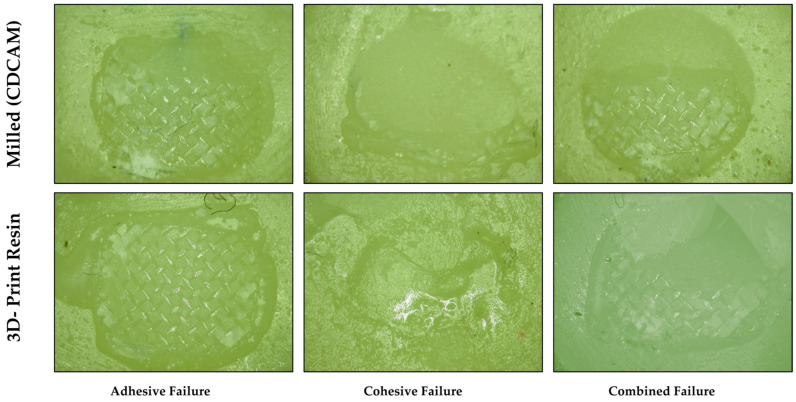
Types of bond failures observed among milled and 3D-printed provisional crown materials, with amount of residual adhesive left deciding the adhesive remnant scores. More adhesive was left in 3D-printed provisional crowns than milled crowns, which also saw a smaller number of adhesive failures.

Adhesive remnant index scores (Figure 4 and Figure 5): The scores that were acquired from the adhesive failure index (ARI) were used to determine the type of adhesive failure that occurred. The extent of the adhesive failure was determined by assigning a score between 0 and 3 to each specimen. Figure 4 shows the frequency distribution of the scores obtained in the subgroups and the scores achieved for each subgroup. The index scores were 0 and 3 for adhesive and cohesive failures, respectively, and 1 and 2 for mixed failures with less than or more than 50% adhesive remaining. Both materials showed entirely different patterns of bond failures depending upon the nature of the material (Figure 5). In milled subgroups, the predominant failure observed was cohesive in nature, whereas in 3D-printed provisional material, the predominant bond failure type occurring in almost all subgroups was adhesive in nature. Printed subgroups showed no evidence of combination failures among their groups, while milled subgroups exhibited more combination failures, particularly with the highest occurrence in the 100-micron particle size subgroup. When compared to milled, there was more leftover adhesive on the 3D-printed provisional crown material irrespective of surface treatment being carried out or the type of surface treatment.

## 4. Discussion

This study was intended to investigate the influence of three different particle sizes of alumina oxide particles upon the shear bond strength of stainless-steel orthodontic brackets to digitally milled and 3D-printed provisional crown materials for long-term clinical use of 20 months or more. The main findings of this study indicate that irrespective of the size of alumina oxide particles, the sandblast surface preparation increases the SBS of stainless-steel orthodontic brackets to both milled and 3D-printed provisional crown material and therefore can be used whenever long-term temporary crowns are indicated for orthodontic or prosthodontic dental patients. The SBS observed for both materials irrespective of surface treatment fulfilled the minimum in vitro criteria of 6 to 8 MPa; however, evidence lacks as to whether such low strengths are sufficient to withstand clinical conditions. Earlier studies investigating the shear bond strength (SBS) of stainless-steel orthodontic brackets bonded to milled and 3D-printed provisional crowns have reported that surface treatment with either coarse or fine diamond burs was associated with a decrease in SBS rather than an increase [1], highlighting the importance of conducting this study.

Material testing methods employed can affect the results obtained. Material mechanical adhesion to surface is generally tested by either using the SBS test or the tensile bond strength tests; however, in terms of direction of applied stress and failure mode produced, these two tests not only differ, but the influence of crosshead speed employed during testing affects recorded values, which should be considered during analysis. The SBS test on orthodontic brackets simulates masticatory stress by measuring the bond’s resistance to forces parallel to the adhesive interface, while the tensile test causes a direct pull-off motion while testing the bond resistance to perpendicular forces. While in tensile tests, the stress distribution is more homogenous, but the alignment and specimen shape impact the final results. Increased crosshead speed during testing may raise SBS readings due to resin adhesives’ viscoelasticity, which stiffens with quicker loading. However, crosshead speed has little to no statistically significant effect on bond strength within the standard testing range (0.1–10 mm/min) because deformation mostly affects ductile materials rather than brittle interfaces [48].

Our primary finding reveals that all subgroups of milled PCM exhibited higher SBS with stainless-steel orthodontic brackets. These findings are therefore in agreement with Hassan NN et al. [1], who reported milled PMMA pucks to have higher SBS than 3D-printed when no surface treatment was performed. The values obtained for 50-micron particles in both milled and 3D-printed provisional material in her study, however, are much lower than those obtained in our study. These can be explained due to the differences in the methodology and tests employed. Digital impression technology is used in both milled and 3D printing technology for recording tooth anatomy and dental cast surfaces, offering significant advantages in storage, replication, transportation, and patient comfort, and overcoming processing errors associated with the fabrication of indirect restorations [25,31]. The differences in functional properties and longevity of restorations are better understood by their relative chemical constituents and their effect on the polymerization process, which differs between the milled and 3D-printed resins. The milled PMMA resin block consists of a polymer matrix and filler, which are mixed and polymerized before curing (prepolymerized). Cracks can occur but can be overcome by blending organic–inorganic composite particles (1–50 microns), preventing air bubble formation [49]. Spherical inorganic composite particles prevent air bubble formation in PMMA CADCAM blocks [50]. These particles, with a diameter of 1–50 μm and 5–60% mass content [51], are suitable for CAD/CAM resin blocks due to their safety, high transparency, and easy refractive index control [49,50]. Polymethyl methacrylate (PMMA) is a preferred polymer due to its ease of polymerizability. Due to flexible timing and simplicity, photopolymerization and thermal polymerization are favored [50]. From manufacture to therapeutic application, each polymerization method has its respective advantage and disadvantage, which needs to be considered before selection. Thermal polymerization promotes homogeneous polymerization from the inner to the outer surface, minimizing interface shrinkage and preventing PMMA block porosity. Free-radical polymerization enhances the polymerization rate while also increasing the propagation as well as the termination rate constant, while high-pressure polymerization increases the mechanical strength and number of higher-molecular-weight polymers. Cutting PMMA resin blocks may contain fillers (other than primary), polymerization initiators, inhibitors, fluorescent agents, UV absorbers, antioxidants, colors, antibacterial agents, and X-ray contrast agents [49,50]. On the other hand, the dental photopolymerizable composition for 3D printers consists of a methacrylate-based polymerizable monomer with a urethane and non-urethane structure, a filler, and an initiator for polymerization [52,53]. Urethane dimethacrylate (UEDMA) is the main component of PMMA 3D print inks, with its chemical reactivity and multiple rotatable bonding abilities [54]. Its chemical reactivity allows it to donate two hydrogen bonds while accepting eight at the same time. The ink’s properties include low viscosity, lower stability, thermal reactivity, and balanced thermoset and thermoplastic properties only after curing [52]. Tetrahydrofurfuryl methacrylate (THFMA), another monomer, is a significant component of PMMA 3D printing ink [55], with its rotatable and covalently bound unit. Ionization occurs when a high-energy electron hits a molecule, resulting in a molecular ion that can break up into smaller fragment ions and neutral bits. A printing resin ink with porosity reduces mechanical qualities (flexural strength, modulus, impact strength), density (prosthesis weight), and clinical performance [52,54]. SBS values vary between different bracket materials (ceramic, metal, and polycarbonate) and restoration surfaces (ceramic, composite, natural tooth, and amalgam) [1,23,56].

The SBS values obtained for 3D-printed PCM (Asiga Dentatooth) in this study with no surface treatment are similar to those obtained in a recent study by Choi Y et al. [57]; however, the SBS values obtained in their study for sandblasting are closer to the ones obtained for 25-micron particle size in our study. The higher SBS bond values obtained in our study are primarily due to the calibration and optimization of the printer prior to printing, which has been observed to improve functional properties of 3D printing [40]. Sayed ME et al. [25] observed that most studies comparing milled PMMA PC with 3D-printed resin did not optimize their 3D printers, as most were performed at manufacturer settings. Another reason for lower bond strength in their study is differences in brackets; whereas our results were obtained for stainless-steel brackets, they used ceramic brackets. Their study, despite lacking thermocycling, utilized provisional crowns instead of disc specimens to match the bracket’s convex contour, thereby increasing the SBS [57]. DentaTooth, used in restorations and prostheses, undergoes polymerization, accumulating monomers and pigments, which later form pores, reducing their physical properties (flexural strength) and allowing for further diffusion [58,59]. Three-dimensionally printed resins generally improve in properties as they age due to conversion of unpolymerized monomers; however, because bracket bonding was performed prior to aging in our study, the lower SBS values can be attributed to a higher quantity of unpolymerized monomers at the time of bonding brackets.

Influence of surface treatment: Our study results that show improved SBS after surface treatment, especially sandblasting using alumina oxide particles of different sizes, further substantiate those studies that have found similar results [1,4,22,30]. Goymen M et al. [21], however, found no surface type influence on bond strength between self-cured and composite resins after 500 cycles, a clinically equivalent time of less than a month. Three-dimensionally printed resins with low polymerization degrees produce unreacted monomers, resulting in high water sorption. After thermocycling, the stabilization of water sorption and the increase in flexural strength result from the crosslinking of multiple monomers and the presence of mineral fillers, which help prevent crack development. This explains the durability of the strength of the orthodontic bracket bond to printed resin in our study. This study also found high SBS values in milled provisional resins, which is primarily due to their basic composition, which includes prepolymerized PMMA blanks with less porosity, high surface hardness, and less chance of water sorption. Longer polymer chains in prepolymerized PMMA pucks increase free monomer conversion, reducing hydrolytic degradation. Low water sorption, solubility, and shrinkage rates contribute to improved clinical performance in the presence of saliva and humid conditions. Our SBS values observed for milled provisional resin material, however, may be considered much higher than those obtained in a few other studies [19,20], which observed SBS values that were not higher than 5.95 MPa. The primary differences stem from the use of different materials and methods, as well as the use of separate tools for surface treatment simulation. The SBS of composites [22,30], polymethyl methacrylate [21,22,60], polyethyl methacrylate [1,22], acrylic resin [22,61], and polycarbonate [22] has been observed to be increased by sandblasting. The average surface roughness after sandblasting and surface roughening in acrylic resins [duralay] was reported by De Almeida JX et al. [60] at 18.04 mpa and 22.64 mpa, respectively. Sandblasting a composite PC resulted in an increase in the SBS value, as observed by Shahin SY et al. [62]. Alumina particles traveling at high speeds penetrate the surface, roughening it and simultaneously forming tiny depressions, cavities, or valleys; the unpenetrated surface, on the other hand, acts as a raised peak, thereby increasing the surface area and producing a mechanical interlocking mechanism that the adhesive can use to cling to the substrate [17]. This also explains why less bond strength was observed with the 100-micron particle size than with the 50-micron particle size. Larger particles create larger craters, while smaller particles create smaller craters on the surface. Depending upon the area of the surface, larger craters will be fewer in number than smaller craters over a specified area. With smaller particle sizes, creating more craters, the number of interlocks increases. When using 25-micron particle sizes, the resulting craters are likely too small to accommodate both the bonding agent and adhesive, which leads to a reduction in bond strength. Another possible explanation for lower bond strength with a lower particle size is that residual alumina particles may remain on the surface, which directly affects the effective contact area for bonding agent. The residual alumina particles later detach during thermocycling, thus reducing the SBS over time. However, it seems unlikely that any residual alumina particle can adhere to resin surface without any adhesive. While it may seem that differences in sand particle sizes will have different surface energies, a recent study found no significant variations in surface energies between particle sizes ranging from 20 to 125 microns [63]. The surface energies reported were in the range of 76.09 to 76.42 mN/m for quartz. They attributed the lack of differences to the fact that surface energy was primarily a material property rather than a size-dependent one and would alter only if surface chemistry or morphology changes. In our case, all three particle sizes had the same morphology (spherical); therefore, changes attributed to differences in surface energies can be ruled out. The kinetic energy of various sand particles in sandblasting is mostly determined by their mass and velocity upon surface impact. The energy of individual particles is essential for the efficacy of surface cleaning, coating removal, or texturing, since increased kinetic energy signifies a more forceful impact and enhanced material removal potential. Particle kinetic energy is influenced by factors such as particle mass, velocity, and particle shape and morphology. Heavier particles carry more energy, while velocity is influenced by air pressure and nozzle design. Since three different particle sizes were used, their kinetic energy would probably affect the outcome, which should be the focus of further in-depth research.

While mechanical properties like toughness are sometimes discussed in relation to restorative materials’ durability, our study evaluated orthodontic brackets’ adhesive performance to CAD/CAM provisional materials, and not their intrinsic resilience. According to ISO/TS 11405:2015 and orthodontic bonding protocols [64], the SBS test evaluates the highest stress needed to debond the adhesive interface. SBS is the most appropriate and most extensively used measure for bracket adhesion in vitro. Shear bond strength is a failure load at the adhesive connection, not energy absorption of the bulk or interfacial material.

Clinical implications: Our study results have specific clinical implications which clinicians should adhere to while treating such cases. Both milled and 3D-printed provisional crowns should undergo mechanical surface treatment in the form of sandblasting whenever clinicians desire provisional crowns to stay functional up to at least 18 months. Sandblasting using 50 and 100 microns are advisable, while no surface treatment and 25 micron alumina sandblasting are not recommended. For anterior teeth, milled provisional crowns have advantages over 3D-printed crown since the removal of orthodontic brackets does not leave any residue, which would not only save clinical time but also maintain crown aesthetics.

Strengths and limitations: Both milling and 3D printing are digital technologies that have the potential to overcome many drawbacks of vintage materials and techniques. What makes this research stand out is that it looks at two popular digital technologies that have already had an effect on the dental industry. Despite the small sample size, this study adheres to a fairly lengthy aging period, which is generally considered clinically desirable. Despite its limited merits, this study does have many limitations, which include its in vitro design, which does not actually mimic clinical conditions, using a single orthodontic bracket material (stainless steel) while not exploring other bracket materials (plastic and ceramic). Also, it only examined a subset of the adhesives and brackets on the market and did not test as many milled and printed PCs as are being marketed. Budgetary restrictions primarily cause these limitations; so, we recommend further research that would investigate other bracket materials like plastic and ceramic, bracket adhesives, bracket designs and various other brands of milled and 3D-printed provisional crowns.

## 5. Conclusions

Within the limitations of this in vitro study, it may be concluded that both the milled and 3D-printed provisional restorative materials investigated in this study demonstrated durable bonding under simulated in vitro aging conditions. The bracket-to-provisional crown bond can be further enhanced by mechanical surface treatment in the form of sandblasting using alumina oxide, with the highest bond strength achieved with a 50-micron alumina particle size. The effect of sandblasting the surface increases the shear bond strength between stainless-steel brackets and milled/3D-printed provisional crown material, regardless of particle size.

## Figures and Tables

**Table 1:** List of materials, instrumentation and manufacturer.

3D-print resin ink (PC)	Asiga Dentatooth Alexandria, Australia	Lot number: MO/16020 [Class II(a)] Composition: Photopolymerized Methacrylate resin: [7,7,9 trimethyl-4,13-dioxo3,14-dioxa-5,12diazahexadecane-1,16diyl bismethacrylate, tetrahydrofurfuryl methacrylate, and diphenyl phosphine oxide Properties: flexural strength (70 to 120 Mpa), modulus of elasticity (3.5 to 4.2 Gpa), fracture resistance (900 to 1400 N). Post processing–light cure Colours: A1, A2, A3, B1, B2, B3 Curing: 4000 flashes (2 × 2000 flashes each side)
CopraTemp	CopraTemp WhitePeaks Dental Solutions GmbH, Wesel, Germany	Lot number: P10690 CADCAM PMMA blanks (shade A1); Plaque resistant Composition: Polymethylmethacrylate pigments Uses: long-term temporary/permanent restoration, splints, preparation guides and denture bases. Flexural strength (80 to 130 Mpa), Modulus of elasticity (3 Gpa), fracture resistance (1100 to 1660 N), Transparent/clear, classic pink, veined pink or precolored in the dentin colors A1, A2, A3 and B1
3D Printing Machine	Asiga Pty Ltd., Alexandria, Australia	Serial: 70B3D5362C6A Model: PN01233 Layer thickness 50 µm Post-Curing Machine-Asiga Flash (Wavelength: 405 nm)
Assure Plus	Reliance Orthodontic Products, Itasca, IL, USA	All Surface Bonding Resin (light cure) Bis-GMA, ethanol, MDP, HEMA
Transbond XT	3M Unitek, Monrovia, CA, USA	Light cure adhesive paste Silane treated quartz (70–80% in weight), bisphenol A diglycidyl ether dimethacrylate, bisphenol A bis(2-hydroxyethyl ether) dimethacrylate, silane treated silica, diphenyliodonium
DGShape Five Axis milling machine	Roland DGA, Irvine, CA, USA	DWX-52D Series (5-axis milling) Operating speed XYZ axis: 6 to 1800 mm/min Spindle speed 6000 to 30,000 rpm Automatic disc changing mill
Thermocycling machine	Model 1100, SD Mechatronik, Bayern, Germany	Alternate immersion in warm followed by cold liquid simulates high temperature changes Warm bath temperature: 25 to 55 °C Cold bath temperature: 5 to 15° C Exposure time—30 s, open air—10 s
Benchtop 3D Scanner	MEDIT Model MD-ID0300, Medit Corp, Seoul, Republic of Korea	Scans full-arch in 8 s 5.0 MP cameras
Imaging powder	VITA CEREC, VITA, Bad Sackingen, Germany	Powder scan spray

**Table 2:** One-way ANOVA (Kruskal–Wallis) rank test results for shear bond strength median values between orthodontic brackets to milled (Copratemp) and 3D-printed (Asiga) provisional restorative material after surface treatment and aging.

Variable 1	Variable 2	Subgroup	N	Median	IQR	Minimum	Maximum	MRS	H Statistic	*p* Value
Material	Surface Treatment	Codes		Mpa						
Milled (Gp M) (N = 40)	No Treatment	MC	10	14.98	2.72	12.15	16.91	20.65	65.0397	0.0000 *
	Alumina oxide (25 µm)	M25	10	16.13	2.71	14.29	19.54	35.70		
	Alumina oxide (50 µm)	M50	10	23.10	2.30	20.50	26.46	72.90		
	Alumina oxide (100 µm)	M100	10	20.00	2.36	17.10	22.39	57.60		
3D-printed (Gp P) (N = 40)	No Treatment	PC	10	12.8	2.74	10.41	14.41	8.95		
	Alumina oxide (25 µm)	P25	10	15.08	1.55	12.50	16.71	22.6		
	Alumina oxide (50 µm)	P50	10	20.72	2.31	17.71	23.88	61.0		
	Alumina oxide (100 µm)	P100	10	17.99	3.45	15.43	20.02	44.6		
Milled (Gp M) (N = 40)	No Treatment	MC	10	14.98	2.72	12.15	16.91	7.4	31.8648	0.0000 *
	Alumina oxide (25 µm)	M25	10	16.13	2.71	14.29	19.54	14.4		
	Alumina oxide (50 µm)	M50	10	23.10	2.30	20.50	26.46	34.7		
	Alumina oxide (100 µm)	M100	10	20.00	2.36	17.10	22.39	25.5		
3D-printed (Gp P) (N = 40)	No Treatment	PC	10	12.8	2.74	10.41	14.41	6.9	31.4283	0.0000 *
	Alumina oxide (25 µm)	P25	10	15.08	1.55	12.50	16.71	14.9		
	Alumina oxide (50 µm)	P50	10	20.72	2.31	17.71	23.88	33.9		
	Alumina oxide (100 µm)	P100	10	17.99	3.45	15.43	20.02	26.3		

Abbreviations: Gp = group; N = number of specimens; IQR = Interquartile range; H = difference between two or more groups of an independent variable on a continuous dependent variable; *p* = probability value; MRS = mean rank score; Mpa = megapascals. Interpretation of groups: M = milled, P = printed, C = control (no surface treatment); 25, 50 and 100 = alumina oxide particle sizes. Test employed—one way ANOVA on ranks (Kruskal–Wallis H test); statistical interpretation: level of the degree of significance was determined on the value of *p* < 0.05; * = significant.

**Table 3:** Tukey’s HSD (honestly significant difference) post hoc pairwise comparison showing overall interactive differences in the sample means between milled (Copratemp) and 3D-printed (Asiga) provisional restorative material using three alumina oxide particle sizes.

Subgroups	Parameters	MC	M25	M50	M100	PC	P25	P50	P100
MC	MRD		−15.05	−52.25	−36.95	11.7	−1.95	−40.35	−23.95
	‘*p*’ value		0.1476	0.0000 *	0.0003 *	0.2602	0.8512	0.0001 *	0.0211
M25	MRD	−15.05		−37.2	−21.9	26.75	13.1	−25.3	−8.9
	‘*p*’ value	0.1476		0.0003 *	0.0350	0.0100	0.2075	0.0149	0.3918
M50	MRD	−52.25	−37.2		15.3	63.95	50.3	11.9	28.3
	‘*p*’ value	0.0000 *	0.0003 *		0.141	0.0000 *	0.0000 *	0.2522	0.0064
M100	MRD	−36.95	−21.9	15.3		48.65	35	−3.4	13
	‘*p*’ value	0.0003 *	0.0350	0.141		0.0000 *	0.0007 *	0.7435	0.211
PC	MRD	11.7	26.75	63.95	48.65		−13.65	−52.05	−35.65
	‘*p*’ value	0.2602	0.0100	0.0000 *	0.0000 *		0.189	0.0000 *	0.0006 *
P25	MRD	−1.95	13.1	50.3	35	−13.65		−38.4	−22
	‘*p*’ value	0.8512	0.2075	0.0000 *	0.0007 *	0.189		0.0002 *	0.0342
P50	MRD	−40.35	−25.3	11.9	−3.4	−52.05	−38.4		16.4
	‘*p*’ value	0.0001 *	0.0149	0.2522	0.7435	0.0000 *	0.0002 *		0.1145
P100	MRD	−23.95	−8.9	28.3	13	−35.65	−22	16.4	
	‘*p*’ value	0.0211	0.3918	0.0064	0.211	0.0006 *	0.0342	0.1145	

Note: Abbreviations HSD = honest significant difference; MRD = mean rank difference; *p* = probability value, * = statistically significant after Bonferroni corrected alpha. Interpretation of groups: M = milled, P = printed, C = control (no surface treatment); 25, 50 and 100 = alumina oxide particle sizes. Test employed—Dunn test with Bonferroni’s corrections; corrected α = α/m = 0.05/28 = 0.001786. Statistical significance: All differences between various subgroups were considered to be statistically significant if the probable *p* value was ≤0.001786.

**Table 4:** Tukey’s HSD (honestly significant difference) post hoc pairwise comparison showing differences in the sample means within milled (Copratemp) and 3D-printed (Asiga) provisional restorative material groups.

Subgroups	Parameters	MC	M25	M50	M100	Subgroups	PC	P25	P50	P100
MC	MRD		−7	−27.3	−18.1	PC		−8	−27	−19.4
	‘*p*’ value		0.1806	0.0000 *	0.0005			0.1264	0.0000 *	0.0002 *
M25	MRD	−7		−20.3	−11.1	P25	−8		−19	−11.4
	‘*p*’ value	0.1806		0.0001	0.0337		0.1264		0.0002 *	0.0292
M50	MRD	−27.3	−20.3		9.2	P50	−27	−19		7.6
	‘*p*’ value	0.0000 *	0.0001		0.0784		0.0000 *	0.0002 *		0.146
M100	MRD	−18.1	−11.1	9.2		P100	−19.4	−11.4	7.6	
	‘*p*’ value	0.0005	0.0337	0.0784			0.0002 *	0.0292	0.146	

Note: Abbreviations HSD = honest significant difference; MRD = mean rank difference; *p* = probability value, * = statistically significant. Interpretation of groups: M = milled, P = printed, C = control (no surface treatment); 25, 50 and 100 = alumina oxide particle sizes. Test employed—Dunn test with Bonferroni’s correction; corrected α = α/m = 0.05/6 = 0.0083. Statistical significance: All differences between various subgroups were considered to be statistically significant if the probable *p* value was ≤0.008333.

## Data Availability

This article presents all important data, while the raw data files are available from the respective corresponding author upon reasonable request.
